# Did Serendipity Contribute to the Discovery of New Antidepressant Drugs? Historical Analysis Using Operational Criteria

**DOI:** 10.31083/AP40037

**Published:** 2025-04-28

**Authors:** Francisco López-Muñoz, Pilar D’Ocón, Alejandro Romero, Domenico De Berardis, Cecilio Álamo

**Affiliations:** ^1^Faculty of Health Sciences - HM Hospitals, University Camilo José Cela, 28692 Madrid, Spain; ^2^HM Hospitals Health Research Institute, 28015 Madrid, Spain; ^3^Neuropsychopharmacology Unit, Hospital 12 de Octubre Research Institute (i+12), 28041 Madrid, Spain; ^4^Department of Pharmacology, Faculty of Pharmacy, University of Valencia, 46010 Valencia, Spain; ^5^Department of Pharmacology and Toxicology, Faculty of Veterinary Medicine, Complutense University, 28040 Madrid, Spain; ^6^National Health Service, Department of Mental Health, Psychiatric Service of Diagnosis and Treatment, “G. Mazzini” Hospital, ASL 464100 Teramo, Italy; ^7^Department of Biomedical Sciences (Pharmacology Area), Faculty of Medicine and Health Sciences, University of Alcalá, 28801 Alcalá de Henares, Madrid, Spain

**Keywords:** antidepressants, history of medicine, psychopharmacology, serendipity

## Abstract

**Objective:**

Given their great importance, as one of the most prescribed types of therapeutic drugs worldwide, we have analyzed the role of serendipity in the discovery of new antidepressants, ranging from selective serotonin reuptake inhibitors to more contemporary developments.

**Methods:**

We carried out a historical analysis of the discovery of new antidepressants, resorting to the original articles published on their development (initial pharmacological and clinical information) and applied an operational criterion of serendipity developed by our group.

**Results:**

Selective serotonin reuptake inhibitors (fluoxetine, fluvoxamine, citalopram, paroxetine, sertraline, and escitalopram), selective dopamine and noradrenaline reuptake inhibitors (bupropion), noradrenaline and serotonin reuptake inhibitors (venlafaxine, milnacipram, duloxetine, and desvenlafaxine), selective noradrenaline reuptake inhibitors (reboxetine), noradrenergic and specific serotonergic antidepressants (mirtazapine), melatonergic agonists (agomelatine), and serotonin modulators and stimulators (vortioxetine, vilazodone, tianeptine) correspond to the type IV pattern. Moclobemide, a reversible monoamine oxidase inhibitor, corresponds to the type II pattern, for which the initial serendipitous findings (i.e., the chance discovery of the inhibitory effects of monoamine oxidase (MAO) whilst being studied for their antihyperlipidemic properties) led to subsequent non-serendipitous discoveries (clinical antidepressant efficacy). Ketamine, a glutamatergic modulator, corresponds to the type III pattern, characterized by a non-serendipitous origin (initial development as an anesthetic agent) leading to a serendipitous observation (the discovery of antidepressant efficacy in individuals illicitly using).

**Conclusion:**

The majority of new antidepressants adhere to a type IV pattern, characterized by a rational and targeted design process where serendipity played no part, except moclobemide (type II pattern) and ketamine (type III pattern).

## Main Points

1. Serendipity is a phenomenon que requires the convergence of accident and sagacity.

2. In pharmacology, purely serendipitous discoveries (type I pattern of imputability) are relatively rare. This pattern has not been found in the discovery of any of the new antidepressants.

3. The development of new antidepressants, from selective serotonin reuptake inhibitors (SSRIs) to the present, has been characterized by a pattern IV, based on rational and systematic research programs and where there is no intervention of serendipity.

4. Moclobemide can be classified under the type II pattern, wherein initial serendipitous findings (the chance discovery of MAO inhibitory effects in a molecule being studied for its antihyperlipidaemic properties) led to subsequent non-serendipitous discoveries (clinical antidepressant efficacy).

5. Ketamine follows a type III pattern, characterized by a non-serendipitous origin (initial development as an anesthetic agent) leading to a serendipitous observation (the discovery of antidepressant efficacy in individuals illicitly using).

## 1. Introduction

The 1950s, renowned in the field of pharmacology as the “golden decade” of psychotropic drugs, ushered in the introduction of the primary groups of agents still used today in treating mental disorders: antipsychotics, anxiolytics and antidepressants [1]. These psychotropic drugs drastically altered psychiatric patient care and provided insight into the neurobiological underpinnings of mental illnesses for the first time in the history, an approach often referred to as “pharmacocentric” [2].

The two main families of antidepressant drugs discovered during this period, tricyclics (TCAs) [3] and monoamine oxidase inhibitors (MAOIs) [4], exhibited significant efficacy in managing affective disorders [1, 5]. However, their substantial clinical contributions were overshadowed by therapeutic challenges primarily associated with tolerability and safety profiles. Consequently, a major challenge in psychopharmacology was overcoming these drawbacks, a feat realized in the 1980s with the clinical introduction of selective serotonin (5-HT) reuptake inhibitors (SSRIs). SSRIs revolutionized depression therapy [5], offering undeniable advantages over classical antidepressants, particularly in terms of safety and tolerability. While zimelidine was the first SSRI introduced, it was subsequently withdrawn from the market, the “new” or “modern” antidepressant era began with the clinical introduction of fluoxetine [6] paving the way for new antidepressant families (see Table 1).

**Table 1.** **Classification of new antidepressants according to action mechanism and historical perspective on their clinical introduction**.

FAMILY	Acronym	Prototype substance	Period
Selective 5-HT reuptake inhibitors	SSRI	Fluoxetine	1975–1990
Reversible MAO inhibitors	RIMA	Moclobemide	1975–1990
Selective DA and NA reuptake inhibitors	SDRI	Bupropion	1975–1990
NA and 5-HT reuptake inhibitors	NSRI	Venlafaxine	1980–2000
Selective NA reuptake inhibitors	SNRI	Reboxetine	1980–2000
Noradrenergic and specific serotonergic antidepressants	NaSSA	Mirtazapine	1980–2000
Melatonergic agonists		Agomelatine	1990–2010
Glutamatergic modulators		Esketamine	1970–2020
Serotonin modulators and stimulators	SMS	Vortioxetine	2000–2020

5-HT, serotonin; MAO, monoamine oxidase; DA, dopamine; NA, norepinephrine; SSRI, selective serotonin reuptake inhibitor; RIMA, reversible monoamine oxidase inhibitors; SDRI, selective dopamine and noradrenaline reuptake inhibitor; NSRI, noradrenaline and serotonin reuptake inhibitor; SNRI, selective inhibitors of noradrenaline reuptake; NaSSA, specific noradrenergic and serotonergic antidepressants; SMS, serotonin modulators and stimulators.

These new drug families, similar to classical drugs in terms of their mechanisms of action, primarily modulate monoaminergic neurotransmission at a synaptic level [2, 5]. Nonetheless, additional antidepressant drug families targeting different neurotransmitter pathways, such as glutamatergics or melatonergics, have also been introduced into clinical practice.

Serendipity is a phenomenon frequently invoked when examining scientific discoveries, including those in pharmacology. Serendipitous discovery is understood as the revelation of something unforeseen, independent of the systematic process leading to the accidental observation [7]. However, the attribution of this phenomenon remains highly contentious, likely due to the semantic ambiguity surrounding the term “serendipity” [8], which has been employed with a wide array of meanings. Traditionally associated with concepts like chance, fortune, randomness, or coincidence (“happy accident”, “pleasant surprise”, etc.), we adopt the approach proposed by the introducer of this term, the English writer, politician and historian Horace Walpole, 4th Earl of Oxford, in his commentary on the classic Persian tale *The Three Princes of Serendip* (1557) [9]. Walpole suggested that serendipity requires the convergence of accident and sagacity, with sagacity being the factor conditioning a serendipitous discovery in the presence of a significant accidental event [7]. This is evidenced by the book *Serendipity*. *Accidental discoveries in Science* [10].

Hargrave-Thomas *et al*. [11] noted that 24% of all commercially available drugs at the time were positively influenced by serendipity during their development, particularly psychopharmacological agents. Confirming this, we found that the discovery of most psychotropic drugs during the 1950s occurred under these parameters of serendipitous influence, including classical antidepressant drugs [7, 12, 13]. To this end, we have established an operational definition of serendipity based on four different attributability patterns [7, 12], allowing us to clarify its role in drug discovery. This paper, following our established approach, examines the role of serendipity in the discovery of new or modern antidepressant drugs.

## 2. Methods

### 2.1 Operational Criteria of Serendipity

**Fig. 1.**
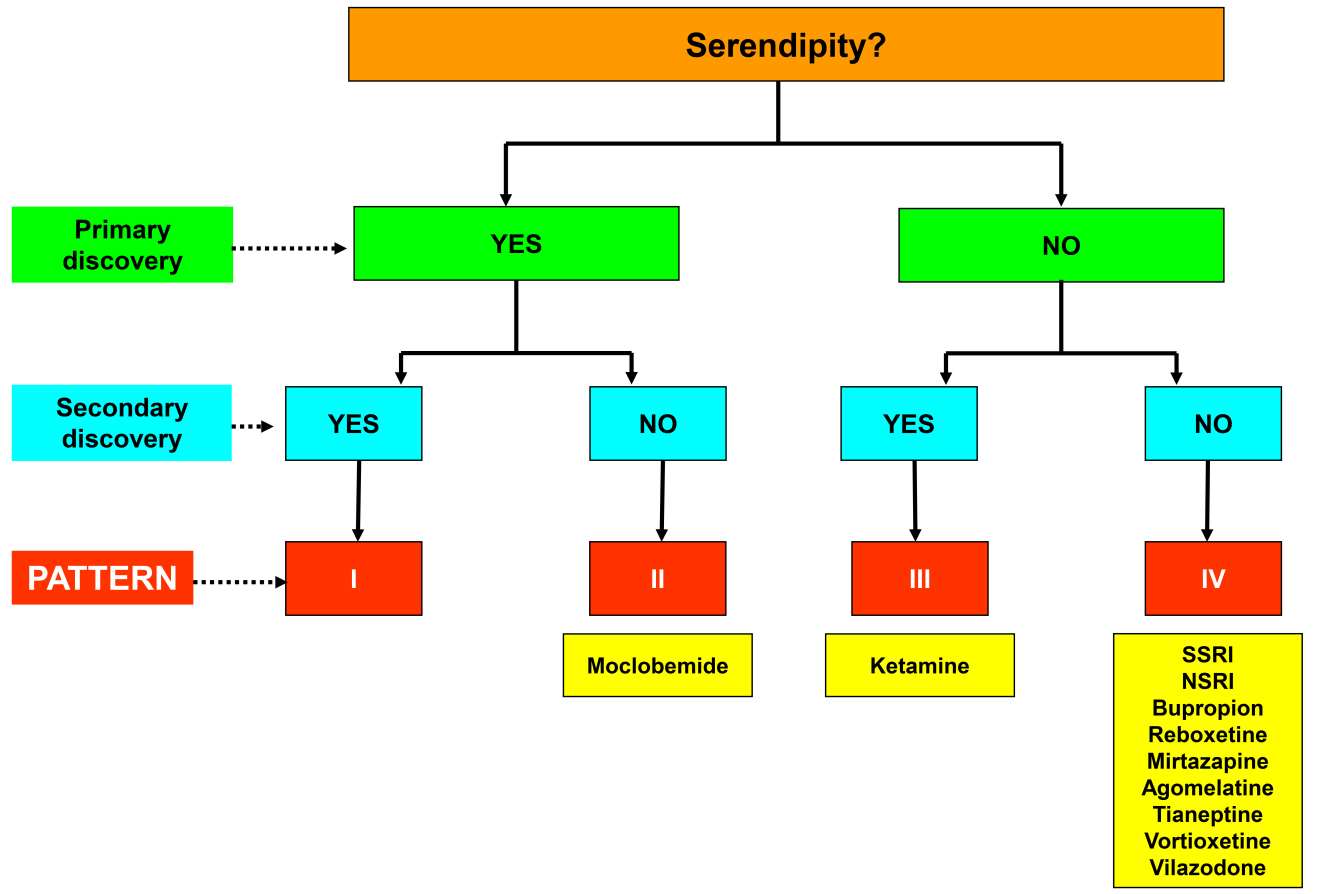
**Diagram of the four patterns of serendipitous attribution in the discovery of pharmacological agents and their application to new antidepressant drugs**. SSRI, selective serotonin reuptake inhibitors; NSRI, noradrenaline and serotonin reuptake inhibitors. Figure adapted from the original created by the authors [7, 13].

In previous research, we have employed a functional characterization of serendipity [7, 12, 13], which involves identifying four distinct patterns of attributing serendipity within the drug discovery process (Fig. 1, Ref. [7, 13]):

(a) Pattern I, encompasses purely serendipitous discoveries.

(b) Pattern II, a modification of the former pattern, delineates chance findings initially leading to planned discoveries devoid serendipitous origins.

(c) Pattern III, comprises discoveries lacking serendipity but subsequently leading to serendipitous findings.

(d) Pattern IV, corresponds to purely non-serendipitous discoveries, occurring outside the realm of chance or unintended accident. This pattern includes discoveries of pharmacological drugs within rational and systematic research programs specifically tailored to produce agents with specific effects on a given pathology.

### 2.2 Archives and Documents on the History of Psychopharmacology

Before applying the attributability criteria, we accessed the original manuscripts containing the initial pharmacological and clinical information on new antidepressant drugs from the various sources:

(a) Primary databases in the biomedical field (Medline, Embase, Scopus).

(b) Documentation resources provided by the pharmaceutical companies distributing the new antidepressants.

(c) Documentation accessible through the International Network for the History of Neuropsychopharmacology (INHN), under the guidance of Thomas A. Ban (Vanderbilt University).

(d) David Healy’s *The Psychopharmacologists* series of interviews de (Arnold – Oxford University Press).

(e) The History of Psychopharmacology collection of the International College of Neuropsychopharmacology (CINP) (*Collegium Internationale Neuro-Psychopharmacologicum*), coordinated by Thomas A. Ban, David Healy and Edward Shorter and published by Animula.

(f) Prof. López-Muñoz’s collection of documents on the history of psychopharmacology.

## 3. Results

### 3.1 Selective Serotonin Reuptake Inhibitors (SSRIs)

The first SSRI synthesized and developed was fluoxetine, considered the prototype molecule of this antidepressant family. Its development stemmed from 1960s studies on TCAs with supported the serotonergic hypothesis of depression by demonstrating the potent inhibition of 5-HT uptake by imipramine and similar agents [1]. A lecture on “synaptosomes” technique, given in 1971 by Solomon H. Snyder from John Hopkins University later influenced fluoxetine’s development. That same year, pharmacologist Ray W. Fuller, an expert in 5-HT research, and biochemist David T. Wong formed a “serotonin-depression study team”, which also included organic chemist Bryan Molloy and pharmacologist Robert Rathbun [1]. In the early 1970s, this team focused on developing molecules that selectively inhibited 5-HT uptake, aiming to create antidepressants without the cardiotoxicity and anticholinergic properties of TCAs [1]. They noted that diphenhydramine and other antihistamines could inhibit monoamine uptake, leading them to synthesized a series of phenoxyphenylpropylamines, including nisoxetine [14]. Among the 55 derivatives in this series, fluoxetine hydrochloride (LY-110140) was identified in 1972 as the most potent and selective 5-HT uptake inhibitor [15]. Fluoxetine’s structure, particularly the p-trifluoromethyl group, was key to its efficacy and selectivity, resulting in fewer adverse effects compared to TCAs [16]. Despite indirect testing methods, studies suggested an increase in extraneuronal 5-HT concentrations [1]. The first publication on fluoxetine appeared in 1974, highlighting its potential utility in studying serotonergic functions and mental disorders [15]. Fluoxetine’s clinical development began in 1980, with initial studies conducted at John Feighner’s private psychiatric clinic in La Mesa (California). By 1983, results demonstrated fluoxetine’s efficacy as an antidepressant with significantly fewer side effects compared to TCAs, marking the advent of a “New Generation of Antidepressants” [17]. Extensive clinical trials between 1984 and 1987 culminated in its approval by the US Food and Drug Administration (FDA) in December 1987.

Following fluoxetine, other SSRIs were introduced, including zimelidine, marketed in 1982, which was withdrawn due to adverse effects (hypersensitivity issues, including fever, myalgias, increased levels of aminotransferases and notably, various cases of neurological complications linked to Guillain-Barré syndrome) [18]. The rest of the SSRIs were marketed later through targeted research strategies. Fluvoxamine was developed by Welle and Claassen [19] in the mid-1970s at Belgium, and marketed in Switzerland in 1983. Citalopram was synthesized in 1972 by chemist Klaus Bøgesø [20] and it was first marketed in Denmark in 1989. Paroxetine, a phenylpiperidine derivative, was discovered in 1975 and patented in the US in 1977. The patent was subsequently transferred in 1980 and was first marketed in Sweden in 1991 [21]. Sertraline’s development began in 1977, led by pharmacologist Kenneth Koe and chemist Willard Welch [22]. This SSRI was launched in the UK in 1990. Finally, in 1997 began the development of escitalopram, (S)-enantiomer of the racemate citalopram [20]. In 2002, escitalopram was launched in Europe and the US and subsequent clinical studies confirmed its superiority over escitalopram, particularly in patients with severe depression [23].

The development of SSRIs marked a shift in psychopharmacology towards rational, directed drug design rather than serendipitous discovery, aligning with our type IV pattern of serendipitous attribution (Table 2).

**Table 2.** **Attribution of serendipity in the discovery of new antidepressant drugs**.

Group/Family	Drug	ATC code	Date of discovery (psychiatric introduction)	Effect/primary properties	Effect/secondary properties	Pattern of discovery
SSRIs	Fluoxetine	N06AB03	1972 (1987)	NS	NS	IV
	Citalopram	N06AB04	1972 (1989)	NS	NS	IV
	Paroxetine	N06AB05	1973 (1991)	NS	NS	IV
	Sertraline	N06AB06	1979 (1990)	NS	NS	IV
	Fluvoxamine	N06AB08	1978 (1983)	NS	NS	IV
	Escitalopram	N06AB10	1988 (2002)	NS	NS	IV
RIMA	Moclobemide	N06AG02	1972 (1992)	S	NS	II
SDRI	Bupropion	N06AX12	1969 (1985)	NS	NS	IV
NSRI	Venlafaxine	N06AX16	(1993)	NS	NS	IV
	Milnacipran	N06AX17	(1996)	NS	NS	IV
	Duloxetine	N06AX21	1986 (2004)	NS	NS	IV
	Desvenlafaxine	N06AX23	(2008)	NS	NS	IV
	Levomilnacipran	N06AX28	(2013)	NS	NS	IV
SNRI	Reboxetine	N06AX18	1984 (1997)	NS	NS	IV
NaSSA	Mirtazapine	N06AX11	1989 (1994)	NS	NS	IV
MA	Agomelatine	N06AX22	1998 (2009)	NS	NS	IV
GM	Esketamine	N06AX27	1962 (2019)	NS	S	III
	Tianeptine	N06AX14	1981 (1989)	NS	NS	IV
SMS	Vortioxetine	N06AX26	2002 (2013)	NS	NS	IV
	Vilazodone	N06AX24	2004 (2011)	NS	NS	IV

NS, non-serendipitous discovery; S, serendipitous discovery; SSRI, selective serotonin reuptake inhibitors; RIMA, reversible monoamine oxidase inhibitors; SDRI, selective dopamine and noradrenaline reuptake inhibitors; NSRI, noradrenaline and serotonin reuptake inhibitors; SNRI, selective inhibitors of noradrenaline reuptake; NaSSA, specific noradrenergic and serotonergic antidepressants; MA, melatonergic agonists; GM, glutamatergic modulators; SMS, serotonin modulators and stimulators. Antidepressant drugs were classified by the Anatomical Therapeutic Chemical (ATC) classification system controlled by the World Health Organization Collaborating Centre for Drugs Statistics Methodology (WHOCC). This system classifies the active ingredient of a drug into groups according to the organ or system on which they have their effect. https://www.whocc.no/atc_ddd_index/?code=N06AX&showdescription=no.

### 3.2 Reversible Monoamine Oxidase Inhibitors (RIMA)

The development of reversible and selective monoamine oxidase A (MAO-A) inhibitors, with moclobemide as the prototype, emerged from efforts to improve the safety profile of classical MAOIs. Moclobemide (Ro 11-1163), a benzamide derivative of morpholine, was first synthesized in 1972 by Pierre-Charles Wyss in Basel (Switzerland) [24]. This synthesis was part of a research programme focused on developing new lipid-lowering agents without antiviral activity. Despite failing to exhibit significant hypolipidemic effects and yielding negative screenings, moclobemide was found to possess intriguing monoamine oxidase (MAO) inhibitory activity in rat studies, prompting a shift towards its development as an antidepressant [24]. *In vitro* studies evidenced moclobemide to be a weak but highly selective MAO-A inhibitor, with a relatively short enzyme inhibition period of 8 to 10 hours [25]. Furthermore, its safety profile was significantly better that of classical MAOIs, with rare instances of problematic hypertensive crises [26].

Clinical trials began in 1977, and moclobemide, was introduced into clinical practice in the UK and Europe in 1992 as the first drug of the new RIMA class. Although it was marketed in various countries and used as a first-line treatment in Finland and Australia, it was never approved in the US.

The serendipitous discovery of moclobemide, originally intended as an antihyperlipidemic, led to its development as a leading example of the new class of antidepressants. This development fits within the type II pattern of our serendipity attribution criteria (Table 2).

### 3.3 Other Monoamine Reuptake Inhibitors

Over the past four decades, numerous new antidepressant drugs have been developed to enhance the basic and clinical profiles of SSRIs. These new drug families, with diverse pharmacodynamic properties, primarily act on the central nervous system (CNS) by modulating monoamine neurotransmission [5]. This includes noradrenaline and serotonin reuptake inhibitors (NSRIs) (venlafaxine, duloxetine, milnacipram, desvenlafaxine, levomilnacipram); selective dopamine and noradrenaline reuptake inhibitors (SDRIs) (bupropion); and selective inhibitors of noradrenaline reuptake (SNRIs) (reboxetine).

Bupropion, originally known as amfebutamone, is a chlorpropiophenone derivative primarily inhibiting dopamine (DA) reuptake, with minimal effect on 5-HT transporters [27]. Developed by Nariman Mehta in 1969 [28], bupropion was introduced to the US market in 1985 but withdrawn in 1986 due to seizure risks, which were later linked to dosage. Reintroduced in 1989 with a maximum daily dose of 450 mg, sustained-release (SR) and extended-release (XL) formulations were approved by the FDA in 1996 and 2003, respectively, to improve patient’s adherence to treatment [29]. Reboxetine, the first, SNRI, was developed at Italy in the mid-1980s and approved in Europe in 1997 [30]. Another potent and selective SNRI is atomoxetine. Initially developed as an antidepressant, it proved more efficacious for the treatment of attention-deficit/hyperactivity disorder (ADHD) and was FDA-approved for this indication in 2002.

Venlafaxine, the first NSRI, was designed to inhibit both 5-HT and norepinephrine (NA) reuptake (with three times greater selective for 5-HT than NA), proving effective with minimal action at other receptors [31]. By optimizing of ciramadol, an opiate analgesic lead, through a three-step synthesis, reducing its chiral centres from three to one, and incorporating an alkylamine pharmacophore, venlafaxine was introduced to the US market in 1993. In 1997, the XL formulation of venlafaxine was also approved for depression [32]. To mitigate potential drug interactions in patients with varying metabolizing capabilities, desvenlafaxine was developed, the primary active metabolite of venlafaxine (O-desmethyl metabolite) [33], and was approved in the US in 2008 [31]. Duloxetine emerged from observations made during research on the molecule LY227942, whose (+)-enantiomer exhibited twice the potency inhibiting 5-HT reuptake compared to the (-)-enantiomer. This antidepressant was introduced to the market in 2004 [34]. Finally, milnacipran, a cyclopropane derivative with 5-HT and NA reuptake inhibitory properties, was approved for use in France in 1996 [35]. Levomilnacipran, the levorotatory enantiomer of milnacipran, was commercialized in 2013 [36].

The development of these drugs was based on rational hypotheses rather than serendipity, reflecting a type IV pattern of drug discovery (Table 2).

### 3.4 Noradrenergic and Specific Serotonergic Antidepressants (NaSSA)

Mirtazapine, known as ORG 3770, is a tetracyclic piperazine-azepinet derived from the antidepressant mianserin. It chemically differs from mianserin by the addition of a nitrogen atom in one of the rings [37]. This atypical tetracyclic antidepressant blocks α_2_ adrenoceptors, 5-HT_2_ and 5-HT_3_ receptors. By antagonizing somatodendritic α_2_ autoreceptors and heteroreceptors located in the pre-synapses, it enhances the firing rate of the neurons and neurotransmitter release [38]. Additionally, its blockade of 5-HT_2_ and 5-HT_3_ receptors helps to moderate some of the known serotonergic adverse effects and improves pharmacological efficacy [39]. The initial data on mirtazapine were published in 1989, and its clinical introduction occurred in the Netherlands in 1994.

As the discovery process of mirtazapine as an antidepressant did not rely on serendipity at any stage, this development aligns with our type IV pattern (Table 2).

### 3.5 Melatonergic Agonists

The hypothesis that led to the discovery of the first melatonergic agent, agomelatine, was based on the manipulation of the circadian rhythm, which is often disrupted in depressive patients [40]. Consequently, a series of naphthalene derivatives were synthesized, and their ability to displace [^125^I]-melatonin in the pituitary gland was investigated [41]. Concurrently, electrophysiological studies were conducted, revealing that one of the derivatives, S20098, acted as an agonist of melatonin receptors. This molecule was selected for further development and named agomelatine [42]. Subsequent studies confirmed that this agent could normalize circadian rhythms in a dose-dependent manner [43] and pharmacological screenings for antidepressant properties yielded positive results [40]. The receptor profile of agomelatine is different from other antidepressant drugs. Agomelatine acts as an agonist of the MT_1_ and MT_2_ melatonin receptors [44], while also functioning as an antagonist of the 5-HT_2⁢C_ receptors [45]. Moreover, it does not inhibit the reuptake of monoamines and lacks the capacity to block other receptors. With its properties as a melatonergic receptor agonist, agomelatine can be regarded as an agent capable of synchronizing distorted rhythms.

Following successful testing of agomelatine in various animal models of depression, the first clinical trial of agomelatine was launched in 2002 by Lôo *et al*. [46] (Hôpital Sainte Anne, Paris), confirming its antidepressant efficacy compared to paroxetine. In March 2006 clinical trials began in the US. However, development for the US market was discontinued in October 2011. Agomelatine was approved for clinical use in Europe in 2009. Despite numerous studies confirming the clinical efficacy of agomelatine in treating depression [47], no further melatonergic drugs have been developed.

The development of agomelatine followed a systematic research program specifically designed to obtain a drug with a melatonin-like profile, devoid of serendipity (type IV pattern of serendipity) (Table 2).

### 3.6 Glutamatergic Modulators

Ketamine (CI-581), a short-acting derivative of phencyclidine, was synthesized in 1962 by Calvin Lee Stevens [48]. It was initially used as a dissociative anaesthetic from 1970 [49] and gained attention for its use in the Vietnam War [50]. Despite its clinical potential, ketamine began being abused recreationally as “Special K” in the mid-1990s, leading to its classification as a Schedule III of the Controlled Substance Act in 1999. Interest in its antidepressant effects emerged in the 1970s [50, 51], but clinical research was delayed for more than two decades by its association with recreational use [1]. A until the first pilot study in 2000 demonstrated that intravenous ketamine at subanaesthetic doses improved depressive symptoms in major depression patients 72 hours after administration [52], leading to a surge in studies confirming its efficacy for treatment-resistant major depression, bipolar disorder and suicidal ideation [53]. Ketamine primarily functions as a non-selective, non-competitive antagonist of the N-methyl-D-aspartate (NMDA) glutamate receptor [54]. Its antidepressant effects are also attributed to other mechanisms such as α-amino-3-hydroxy-5-methyl-4-isoxazolepropionic acid (AMPA) receptor-mediated signalling, increased DA levels in the prefrontal cortex and nucleus accumbens [55]. Following the discovery that, esketamine, the S enantiomer of ketamine, was higher NMDA receptor affinity [51], a double-blind clinical trial showed its rapid antidepressant effects in treatment-resistant depression after intravenous infusion [56]. Subsequently, the possibility of administering low doses of esketamine intranasally was explored [57] and was approved by the FDA and European Medicines Agency (EMA) in 2019 for use in resistant depression in combination with an SSRI or SNRI, with administration monitored in clinical settings [58]. Despite its benefits, esketamine has a higher incidence of dissociative effects and potential abuse risk due to its hallucinatory properties [59].

Tianeptine, discovered by the French Society of Medical Research in the 1960s and patented by researchers Antoine Deslandes and Michael Spedding in 1981 [60], differs from traditional TCAs, as amineptine, by not affecting 5-HT reuptake [61]. Instead, it has a weak action on µ-opioid receptors and modulates glutamatergic mechanisms [62]. Although initially developed for major depressive disorder, its US development was discontinued in 2012 [63]. Tianeptine was later marketed as a nootropic supplement but faced scrutiny due to high abuse potential and legal concerns [64]. This led to the coining of the term “gas station heroin”. Since 2023, products containing tianeptine have been gradually withdrawn.

Ketamine has garnered significant attention in psychiatric research as a prototype for a new generation of antidepressants following the discovery of its profound and rapid effects on depressive symptoms. However, it stands as a clear example of serendipity resulting from a non-serendipitous discovery (type III pattern) (Table 2). Originally developed as an anaesthetic agent, its antidepressant efficacy was stumbled upon coincidental when improvement in depressive symptoms was observed in individuals using the substance as a drug of abuse.

### 3.7 Serotonin Modulators and Stimulators (SMS)

Vortioxetine is a bis-aryl-sulfanyl amine and piperazine derivative, whose rationale and synthesis (Lu AA21004) was detailed in 2002 [65]. It was introduced into the US market in 2013. This compound represents a new class of antidepressants known as SMS or “multi-modal”, due to its high binding affinity and complementary mechanisms of action on several 5-HT receptors and 5-HT transporters. Specifically, vortioxetine acts as a 5-HT_1⁢A_ receptor agonist, 5-HT_1⁢B_ receptor partial agonist, 5-HT_3⁢A_ and 5-HT_7_ receptor antagonist, and a potent 5-HT reuptake inhibitor [66]. Additionally, vortioxetine exhibits significant affinity for DA and NA transporters, being 3 to 12 times more selective for 5-HT transporters, respectively [67].

Vilazodone received approval medical use in the US in 2011. Classified as a SMS or serotonin partial agonist/reuptake inhibitor (SPARI), vilazodone possesses SSRI properties and acts as an activator of the 5-HT_1⁢A_ receptor [68].

The development of SMS drugs was driven by rational planning based on an understanding of the role of serotonergic neurotransmission in affective disorders, with minimal reliance on serendipity (type IV pattern of serendipity) (Table 2).

## 4. Discussion

As articulated by Albert Szent-Györgyi, the discoverer of vitamin C, “a discovery consists of seeing what everybody has seen and thinking what nobody has thought” (p. 57) [69]. Therefore, in line with the original conceptualization, we contend that “*serendipity*” pertains to the discovery of something unexpected or not deliberately sought [7, 12], i.e., a finding that arises without the observer anticipating it.

In pharmacology, purely serendipitous discoveries (our type I pattern of imputability), are relatively rare, contrary to popular belief. Most discoveries exhibit a mixed character (Patterns II and III), blending serendipitous and non-serendipitous elements. Typically, this follows a consistent pattern that starts with an initial serendipitous observation, often encountered in laboratory-based animal research, which subsequently prompts deliberate investigations in a clinical setting (our Pattern II). These circumstances contribute to the existence of the aforementioned discrepancies among authors. Some interpret these findings solely through the lens of chance, positing that the results of clinical trials represent a *continuum *of the initial serendipitous findings and should be regarded as part of a unified discovery rather than separate events. Other authors refer to these patterns as “pseudoserendipity” [10] or “serendipity analogous” discoveries [70].

However, it is evident that serendipity, to varying degrees, played a fundamental role in the initial decades of modern psychopharmacology, particularly in the discovery of the first two families of antidepressant drugs (TCAs and MAOIs) [3, 4, 13]. This process yielded revolutionary outcomes in the realm of mental health, influencing various aspects of socio-health reality. These include the progressive phenomenon of “deinstitutionalization” in psychiatry and the involvement of primary care into mental health services, both mitigating the historical stigmatization associated with psychiatric care. Moreover, this “revolution” spurred scientific advancements, including the formulation of initial biological hypotheses concerning the genesis of mental illnesses, particularly affective disorders [2, 71]. At the nosological level, the introduction of these drugs contributed, to some extent, to shaping new diagnostic criteria. Furthermore, the emergence of classic psychotropic drugs enhanced clinical research by facilitating the synthesis of numerous drugs for mental disorder treatment.

The development of new drugs for affective disorders benefited significantly from the tenets of monoaminergic theories of depression, which dominated scientific discourse in specialized journals during the 1960s and 1970s following the discovery of imipramine and iproniazid. These theories posited a functional deficiency in noradrenergic or serotonergic neurotransmission [2, 5] in specific brain regions as a primary cause of these pathologies [72], paving the way for new families of antidepressant drugs characterized by modulating monoaminergic functionalism.

Hence, the findings of our study appear to affirm that since the 1980s, the development of new antidepressant agents have followed a different dynamic, marked by a systematic and deliberate pursuit of outcomes, with minimal reliance on serendipity (our Pattern IV). Some authors have underscored the role of serendipity in contemporary scientific research in this field. Donald Klein [73], in his article *The Loss of Serendipity in Psychopharmacology*, partly attributes the relative dearth of innovation in psychopharmacology over the past five decades to the absence of serendipity. He advocates for fostering serendipity through structured research environments [73]. In our editorial, we identified several “anti-serendipity” factors that may explain this phenomenon outlined by Klein; (a) the scientific shift towards rational drug design grounded in translational research; (b) reduced time for researchers to observe and engage with patients; and (c) pharmacology’s reliance on clinical trials employing double-blind, placebo-controlled designs as the primary method for demonstrating drug efficacy [74]. Additionally, other authors highlight more specific factors, such as advances in genetic and diagnostic imaging techniques, including nuclear magnetic resonance, combinatorial and computational chemistry, X-ray crystallography, etc. [75].

Over the past four decades, the discovery and identification of new chemical entities (NCE) have predominantly focused on elucidating the molecular targets with which these agents interact. This approach, often termed “targephilia”, by some authors, adopts a reductionist perspective that emphasizes the specific sites of drug action [76]. This paradigm has prevailed in the development of new antidepressant drugs, leading to a practical decline in serendipitous discoveries within this process, as evidenced by the findings of our study. Nonetheless, certain exceptions have been noted, such as in the cases of moclobemide and ketamine. Serendipity played a pivotal role in the discovery of the antidepressant properties of both drugs, as previously mentioned, thereby categorizing them within our type II and III patterns of imputability, respectively.

Despite the considerable clinical advantages of new antidepressants, which are largely attributed to their improved tolerance, safety profile, and greater convenience, several challenges persist in antidepressant therapy. Notable among these challenges are the delayed onset of antidepressant response and the estimated percentage of non-responsive patients, which stands at around 30% [77]. Additionally, inadequate antidepressant response affects 40–50% of patients. This may be influenced by the convergent pharmacodynamic properties of these new antidepressants, which often enhance aminergic function: Examples include NSRI (venlafaxine, duloxetine, milnacipram), NaSSA (mirtazapine), SDRI (bupropion), and SNRI (reboxetine, atomoxetine) [5]. Moreover, some recent antidepressants target non-aminergic receptors, albeit not entirely selectively. For instance, agomelatine, a melatonergic agonist (MT_1_ and MT_2_) also blocks 5-HT_2⁢A_ receptors, thereby facilitating the release of DA and NA in the prefrontal cortex [78]. The antidepressant arsenal has further expanded with vortioxetine, characterized as SMS or “multi-modal”, given its impact on the functionalism of several monoamines. Its primary mechanism involves a combination of SSRI, 5-HT_3_ receptor antagonism and 5-HT_1⁢A_ receptor agonism, thus, affecting monoaminergic pathways as well. Lastly, tianeptine is an antidepressant with a complex mechanism involving glutamatergic functionalism, along with esketamine, which influences opioidergic and DA release in the nucleus accumbens and prefrontal cortex. Therefore, these latter antidepressants also engage monoaminergic mechanisms [62].

Depression is a complex pathology characterized by pathophysiological mechanisms that extend beyond solely aminergic pathways. As a result, research into the mechanisms of action of antidepressants has begun to explore alternative directions. These include investigations into neurotrophic factors such as neurotrophins and brain-derived neurotrophic factor (BDNF), corticotrophin releasing hormone (CRH), glucocorticoids, and various other potential targets as the opioid system, interleukins, substance P, somatostatin, neuropeptide Y, melatonin, nitric oxide, among others. Moreover, studies are delving into intraneuronal molecular signal communication pathways. Therefore, the quest for antidepressants with clinical profiles distinct from conventional ones involves a deeper exploration of depression’s pathogenesis and the use of pathophysiological findings as potential therapeutic targets. In this evolving landscape, serendipity may once again emerge as a relevant factor.

## 5. Conclusion

The discovery of the majority of new antidepressants adhere to a type IV pattern, characterized by a rational and targeted design process where serendipity played no part. This is the case of SSRIs (fluoxetine, fluvoxamine, citalopram, paroxetine, sertraline, and escitalopram), SDRI (bupropion), NSRIs (venlafaxine, milnacipram, duloxetine, desvenlafaxine), SNRI (reboxetine), NaSSA (mirtazapine), melatonergic agonists (agomelatine), and SMS (vortioxetine, vilazodone, tianeptine). However, there are two exceptions: moclobemide and ketamine. Moclobemide, a RIMA, can be classified under the type II pattern, wherein initial serendipitous findings (such as the chance discovery of MAO inhibitory effects in a molecule being studied for its antihyperlipidaemic properties) led to subsequent non-serendipitous discoveries (clinical antidepressant efficacy). Ketamine, a glutamatergic modulator, follows a type III pattern, characterized by a non-serendipitous origin (initial development as an anesthetic agent) leading to a serendipitous observation (the discovery of antidepressant efficacy in individuals illicitly using ketamine).

Since the 1980s, psychopharmacology has been evolving outside the influence of serendipity, but this phenomenon eventually becomes apparent, as has happened in the case of ketamine. However, the results of this work are limited by the data provided by the discoverers themselves in their scientific articles and in their memoirs. In addition, the scientific construct of the serendipity phenomenon itself has varied over time and its role has been interpreted differently by each researcher. Fortunately, the operational criteria of serendipity developed by our group has allowed us to address this issue in a fairly objective and satisfactory way.

## Availability of Data and Materials

All data analyzed in the current study is included in the manuscript.
